# Magnetic Resonance Imaging Techniques for Post-Treatment Evaluation After External Beam Radiation Therapy of Prostate Cancer: Narrative Review

**DOI:** 10.3390/clinpract15010004

**Published:** 2024-12-27

**Authors:** Eleni Bekou, Admir Mulita, Ioannis Seimenis, Athanasia Kotini, Nikolaos Courcoutsakis, Michael I. Koukourakis, Francesk Mulita, Efstratios Karavasilis

**Affiliations:** 1 Medical Physics Laboratory, School of Medicine, Democritus University of Thrace, 69100 Alexandroupolis, Greece; ebekou@med.duth.gr (E.B.); akotini@med.duth.gr (A.K.); ekaravas@med.duth.gr (E.K.); 2 Department of Radiotherapy/Oncology, School of Medicine, Democritus University of Thrace, 69100 Alexandroupolis, Greece; amoulita@med.duth.gr (A.M.); mkoukour@med.duth.gr (M.I.K.); 3 Medical Physics, Medical School, National and Kapodistrian University of Athens, 11528 Athens, Greece; iseimen@med.uoa.gr; 4 Radiology Department, School of Medicine, Democritus University of Thrace, 69100 Alexandroupolis, Greece; ncourcou@med.duth.gr; 5 Department of Surgery, University Hospital of Patras, 26504 Patras, Greece

**Keywords:** prostate cancer, radiation therapy, biochemical recurrence, MRI, radiomics

## Abstract

**Background/Objectives**: This study aimed to investigate the prognostic value of advanced techniques of magnetic resonance imaging (MRI) biochemical recurrence (BCR) after radiotherapy in patients with prostate cancer (PCa). **Methods**: A comprehensive literature review was conducted to evaluate the role of MRI in detecting BCR of PCa patients after external beam radiation therapy. **Results**: National guidelines do not recommend imaging techniques in clinical follow-up PCa. However, in 2021, the European Association of Urogenital Radiology (ESUR), the European Association of Urological Imaging (ESUI), and the PI-RADS Steering Committee introduced the Prostate Imaging for Recurrence Reporting (PI-RR) system. PI-RR incorporates the MRI biomarkers in the post-treatment process. In the last decade, a growing number of clinical researchers have investigated the role of various MRI techniques in BCR. **Conclusions**: The integration of advanced MRI technologies into clinical routine marks the beginning of a new era of BCR with accuracy.

## 1. Introduction

Prostate cancer (PCa) is the second most diagnosed cancer in men and the fourth cause of death in both genders worldwide [1,2]. According to the World Health Organization [3], there were approximately 1.468 million new cases of prostate cancer diagnosed worldwide and nearly 397,000 global deaths from the disease in the year 2022 [2]. Radiation therapy (RT) with or without androgen deprivation therapy (ADT) is one of the main treatment strategies for PCa [4]. However, patients with PCa undergoing radiotherapy have the possibility of biochemical recurrence (BCR) due to local or distant PCa evolution, etc. [5]. BCR is shown in 25% of patients treated with RT with the detection of localized recurrence proven accurately histologically [6,7]. Prostate-specific antigen (PSA) serum in blood is a noninvasive method to control BCR. After RT, BCR can be suspected according to the Phoenix Criteria when an absolute increase in PSA level by 2 ng/mL above the PSA level nadir (i.e., the lowest posttreatment value)occurs [8]. Nevertheless, a false-positive increase in PSA levels can arise, unrelated to tumor recurrence or residual disease [9]. Therefore, other diagnostic modalities can be used to complement the prognosis of PCa recurrence [9].

In the recent decades, there has been increasing interest in magnetic resonance imaging (MRI) to manage prostate cancer recurrence after external beam radiation therapy (EBRT) [4]. Based on the above, this review aims to standardize the MRI protocols for evaluating PCa using MRI before and after treatment with EBRT.

### 1.1. The Role of PSA on Follow-Up

Measurement of PSA is the starting point of follow-up after RT [4]. The PSA test is a blood test that measures the amount of prostate-specific antigen (PSA) in the blood. PSA is a protein produced by cancerous and normal tissue in the prostate gland. The primary location of PSA is in semen, but small amounts of it circulate in the blood. Elevated levels of PSA may suggest the presence of prostate cancer. However, hyperplasia or prostatitis can increase PSA levels. Therefore, a high PSA score could be complicated [5,6].

The PSA level also defines treatment success or failure. PSA failure after primary RT, with or without short-term hormonal manipulation, is ‘any PSA increase >2 ng/mL higher than the PSA nadir value, regardless of the serum concentration of the nadir’, according to RTOG-ASTRO Phoenix Consensus Conference. A rise in PSA levels following treatment for prostate cancer indicates BCR [4]. Thus, physicians should carefully interpret PSA arising based on EAU BCR risk groups [2]. Clinicians should take a PSA test every three months during the first year and every six months after the first year [7].

However, there is evidence that doubts the determinant role of PSA on BRC. The 70–80% of patients with elevated PSA levels (>4 ng/mL) do not come up again with PCa [8]. Additionally, PSA cannot define local recurrence or distant metastasis [3,9]. Thus, PSA is not considered a crucial modality to determine and evaluate BCR.

### 1.2. The Role of Magnetic Resonance Imaging MRI on Follow-Up

Radiologists have used multiparametric magnetic resonance imaging MRI (mpMRI) since the 1980s in the diagnosis and staging of prostate cancer [10] following prostate imaging—reporting and data system (PIRADSv2.1) guidelines [11]. In radiation oncology, mpMRI offers improved contrast resolution compared to conventional CT and is considered a powerful tool for evaluating treatment response, it is not used in clinical practice [12].

The first suggestion of using MRI in the initial and repeat assessment of men on active surveillance came from the UK National Institute for Health and Care Excellence (NICE) in 2014 [13]. In 2021, a group of international experts from the European Association of Urogenital Radiology (ESUR), European Association of Urological Imaging (ESUI), and PI-RADS Steering Committee published a standardized Prostate Imaging for Recurrence Reporting (PI-RR) system [14].

#### 1.2.1. Prostate Imaging for Recurrence Reporting (PI-RR) System

The PI-RR system is a 5-scale scoring system that evaluates the level of suspicion of PCa recurrence based on mpMRI evidence over the whole prostate gland.

**Figure 1:**
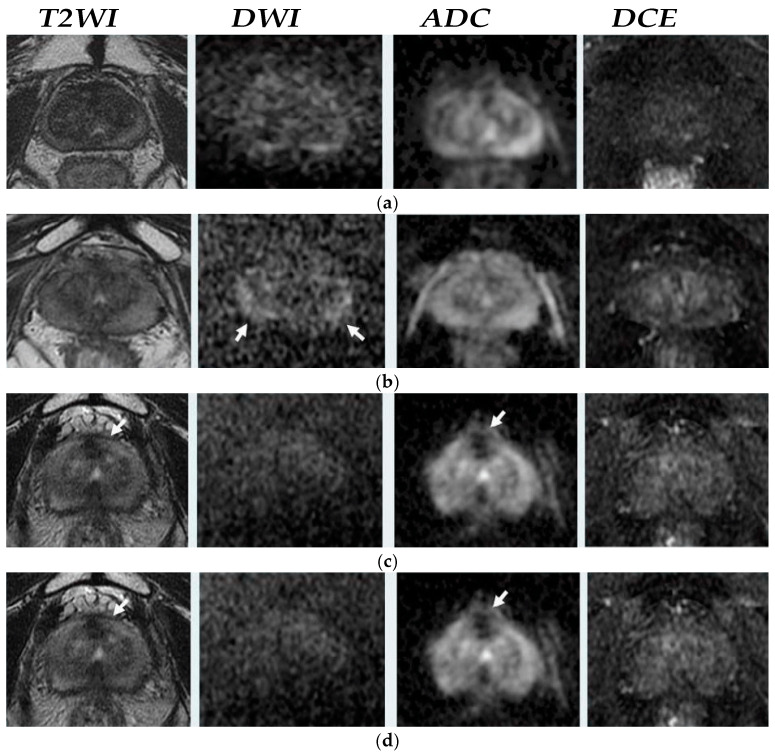

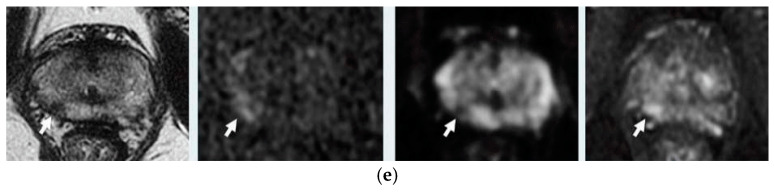
Axial MRI example in post-radiation therapy (RT) by Pecoraro et al. [16]. (**a**) MR images in 82-year-old man with biochemical recurrence (BCR) (prostate-specific antigen–PSA = 0.09 ng/mL) without suspicious foci of local recurrence, Prostate Imaging for Recurrence Reporting PI-RR 1. (**b**) MR images in an 85-year-old man with BCR (PSA = 0.09 ng/mL), show diffuse hyperintensity ta high-b value diffusion-weighted imaging DWI (arrows), PI-RR 2. (**c**) MR images in a 68-year-old man with BCR (PSA = 5.2 ng/mL) indicate hypointense focus at T2weighted Imaging (T2WI) (arrow), low intensity on the apparent diffusion coefficient (ADC) map (arrow) and no hyperintensity at DWI and no early enhancement on dynamic contrast enhancement (DCE), PI-RR 3. (**d**) MR images in an 83-year-old man with BCR (PSA = 3.1 ng/mL) with similar findings with previous patients and hyperintensity at high-b value DWI and ADC map and not completely matching the enhancement area in DCE, PI-RR 4. (**e**) MR images in a 73-year-old man with BCR and PSA = 2.4 ng/mL with the same PCa recurrent evidence with PI-RR 4, however, observed matching the enhancement area on DCE image, PI-RR 5.

The malignancy is scored by radiologists assessing T2-weighted (T2WI), diffusion weighted imaging (DWI), and diffusion contrast enhancement (DCE) images after RT. A score of 1 corresponds to a lesion with a lower risk of recurrence; 2, a low risk of recurrence; 3, indistinct or uncertain risk of recurrence; 4, a high risk of recurrence; and 5, a very high risk of recurrence [15]. Figure 1 represents examples of PI-RR score in axial post-radiation MR images from the Pecoraro et al. study [16]. PI-RR not only evaluates PCa recurrence but also represents recommendations for the management of patients with recurrent prostate cancer by improving diagnostic performance and personalized treatment for each patient [17].

PI-RR systems have proven adequate for patients with BCR in three multi-reader studies. Pecoraro M. et al., in patients treated with RT, indicated a sensitivity of 71–81%, specificity of 74–93%, positive predictive value (PPV) of 71–89%, negative predictive value (NPV) of 79–86% and excellent intrareader agreement with an intraclass correlation coefficient ICC = 0.87 [16]. Bergaglio C. et al. in the RT group observed sensitivity of 59–83%, specificity of 87–100%, an accuracy of 80–88% and lower ICC = 0.74 due to a marked difference in experience among readers from 3 to 10 years [18]. The most recent research by Franco N.P et al. has comparable results with previous studies despite having two PI-RR cut-off values [19]. With the first cut-off PI-RR ≥ 3, sensitivity, specificity, PPV and NPV ranges were 79–87%, 64–86%, 95–98% and 33–46%, respectively. On the other hand, reduced diagnostic accuracy was obtained with the cut-off PI-RR ≥ 4 (sensitivity 74–80%, specificity 64–86%, PPV 94–98%, NPV 28–36%). The lower NPV in both PI-RR cut-off values may be related to the asymmetrical distribution of the study population. All the above results provide PI-RR score systems a structured, reproducible, and accurate assessment of local recurrence after definitive therapy for prostate cancer.

#### 1.2.2. MRI Sequences Suggestions on EBRT

According to EUA Guidelines, follow-up of localized prostate cancer is typically not recommended unless patient belongs in the EAU high risk group (interval to biochemical failure ≤ 18 months or biopsy ISUP grade group 4–5) which could indicate potential recurrence and thus necessitate further diagnostic evaluation [2,20]. The application and selection of imaging techniques are desired by institutional experience and collaborative decision-making between radiologists, urologists, and radiation oncologists if there is the possibility that the findings will affect treatment decisions [2,20]. The scientific community is interested in the use of mpMRI in the evaluation of recurrent or residual disease.

MRI is a non-invasive imaging modality that generates images of soft tissues, providing structural information on patients’ anatomy without the risk of ionizing radiation [20]. MpMRI combines the anatomic information from T1 and T2WI sequences with functional information from DWI and DCE [11]. Table 1 summarizes the useful sequences for BCR failure, which are analyzed in the following sections with the advantages and disadvantages of each sequence.

#### 1.2.3. Multiparametric MRI Suggestion on Recurrence

External beam radiotherapy (EBRT) causes atrophy, inflammation, and fibrosis within the target area, resulting in a reduced prostate gland size and a diffuse T2 hypointense with decreased contrast between the treated tumor and the background prostate tissue on MRI [14]. These post-radiation inflammatory changes lead to false-positive interpretations [14]. MRI imaging should be applied cautiously after the first three months as a baseline to evaluate recurrence or residual disease [8].

##### T2—Weighted Imaging T2WI

On T2WI MRI, the architecture of the prostate, including the peripheral zone, transition zone, prostatic urethra, prostatic capsule, and seminal vesicles, is clearly defined. This imaging technique is also beneficial for detecting which have a shorter “T2 relaxation time” in contrast with normal glandular tissue, making them appear darker than in the peripheral zone and slightly brighter and more homogeneous in the transition zone [21].

In case of local recurrence, T2WI appears as a hypointense nodular lesion mass that may exhibit a capsular bulge [14,22] (Figure 1). This lesion is relatively hypointense compared with treated prostatic tissue due to the rapid growth of the tumor in contrast to the atrophic tissue [9,22]. Cancer most commonly recurred at the original site of the primary tumor [23]. In addition, with background signal changes within the prostate, T2WI was rendered with limited diagnostic accuracy [15,24].

##### Diffusion Weighted Imaging DWI-MRI

DWI is a crucial element of mpMRI that assists in the response to treatment in various cancers [25]. DWI measures the random Brownian motion of water molecules within tissue. In solid tumors, the cell density is typically high, restricting this movement. The degree of diffusion restriction is through the apparent diffusion coefficient (ADC) derived from DWI [26]. After effective treatment, the tumor’s cellular density typically decreases due to cell death. Cell death diminishes the water molecule movement, resulting in increased diffusion and elevated ADC values [27]. An increasing ADC value post-treatment indicates a positive treatment response [8].

**Figure 2:**
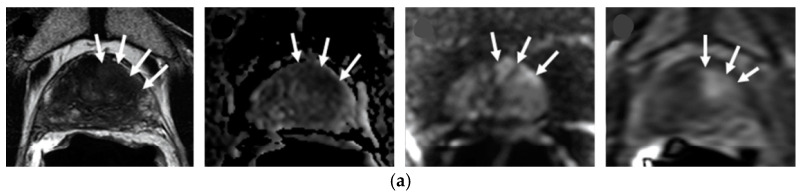

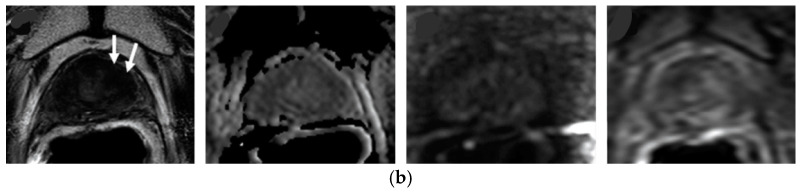
A 55-year-old man who was treated with external beam radiotherapy. Pre-treatment prostate-specific antigen—PSA level was 7.96 ng/mL, and 1 year after treatment, PSA level was decreased in 0.76 ng/mL. (**a**) Original tumor on transition zone (arrow) on Axial T2weighted Imaging (T2WI), apparent diffusion coefficient (ADC), b-2000 diffusion weighted (DW) and diffusion contrast enhancement (DCE) images (from right to left) (**b**) The 1 year posttreatment follow-up observed decreased prostate size, lower DWI signal and no focal enhancement on DCE imaging.

In cases of suspected local recurrence, cancerous tissues often restrict water diffusion due to increased cellularity. This restriction increases the signal intensity on DWI and reduces the ADC values on the ADC map generated from DWI [21,27]. Therefore, recurrent prostate tumors often appear as areas of high signal intensity on DWI and have low ADC values, which contrast with the signal characteristics of the normal prostate or benign post-treatment changes [25,27] (Figure 1 and Figure 2).

##### Dynamic Contrast-Enhanced DCE-MRI

The sensitivity of DCE-MRI to vascular characteristics makes it a potential biomarker for assessing the therapeutic response in prostate cancer. Prostate cancer typically stimulates new blood vessel growth—a process called angiogenesis—leading to a denser vascular network with increased vessel permeability compared to normal tissue. The heightened angiogenic activity results in a more rapid and noticeable uptake of intravenous gadolinium-based contrast media used in DCE-MRI and is considered an earlier and more significant enhancement of dynamic T1-weighted images relative to normal prostate tissue [22]. Figure 2 illustrates a case of post-treatment DCE images compared to pre-treatment DCE images by Gaur and Turbey study [9].

Following RT for PCa, changes in blood flow and perfusion detected via DCE-MRI are indicative of the treatment’s effectiveness. An increase in blood perfusion seen on DCE-MRI post-radiotherapy can denote a positive response to treatment [28]. Thus, DCE MRI is particularly essential as it can show early enhancement and wash-out patterns characteristic of recurrent tumors, which helps to differentiate them from post-treatment changes such as fibrosis or granulation tissue that may appear after radiation treatment [29] (Figure 1 and Figure 2). The main drawback of DCE is the requirement to conduct it at least three months following RT because the inflammatory response to radiation can cause changes in perfusion and blood volume, possibly leading to false results [14].

##### Magnetic Resonance Spectroscopy (MRS)

**Figure 3:**
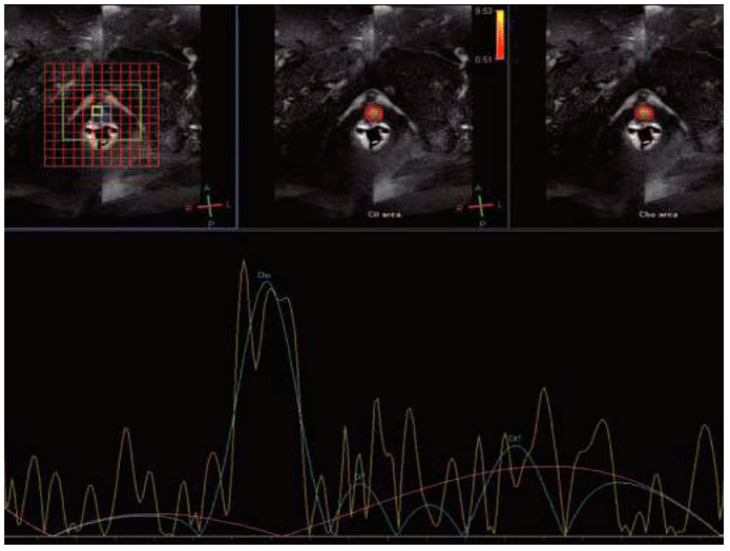
Magnetic resonance spectroscopy showed high levels of choline (cho) levels with increased ratio of choline + creatine(Cr)/Citrate(Cit) = 2.81 in a 60-year-old man with biopsy-approved biochemical recurrence (PSA = 4.5 ng/mL) 4 years after external beam radiotherapy [31].

Magnetic resonance spectroscopy (MRS) can measure specific metabolites within intracellular and extracellular spaces of tissues. In prostate tissue, choline and citrate are two significant metabolites, with citrate representing prostate energy metabolism and choline indicating tumor membrane cellular activity [30]. Following RT, BCR may lead to alterations in cellular function, including a rapid decrease in citrate levels. Choline levels tend to be considerably higher in more aggressive tumors, correlating with the extent of tumor cell proliferation or necrosis [26]. Figure 3 illustrates a case patient with BCR where high levels of Choline were observed in the Roy et al. study [31]. However, the clinical application of MRS is restricted by the long image acquisition time, high cost of exams, and relatively low sensitivity [28].

##### Blood Oxygenation Level Dependent (BOLD) and Tissue Oxygen Level Dependent (TOLD) MRI

Hypoxia plays a crucial role in aggressiveness and treatment resistance to treatment of various cancers, including prostate. Low oxygen levels in cells are associated with increased resistance to radiation therapy [27,28]. Oxygen-enhanced MRI is a non-invasive MR technique that allows the measurement of the tumors’ dynamic oxygenation levels with potential integration into clinical practice [27].

BOLD MRI utilizes the tissue water proton apparent transverse relaxation rate (R2*), which is affected by the concentration of paramagnetic deoxyhemoglobin monomer (Hb).

Thus, the R2* signal can assess oxygen levels in blood and adjacent tissues. However, the transformation of deoxyhemoglobin to oxyhemoglobin, changes in flow, hematocrit and vascular volume influence the R2. In contrast, the longitudinal relaxation rate (R1 = 1/T1) can determine the number of molecules of free oxygen, corresponding to the partial pressure of oxygen (pO_2_), using a technique referred to as tissue oxygen-level-dependent imaging (TOLD) [29]. Both BOLD and TOLD imaging techniques showed capable biomarkers for the prediction of failure outcomes of RT. However, the current research is limited to studies on rat prostate glands, and further investigation is required [32].

##### Intravoxel Incoherent Motion MRI

Intravoxel incoherent motion (IVIM) is a technique that provides information about perfusion and diffusion without the use of contrast agents. Applying an exponential fit of the DWI signal would separate the signal of flowing water in the capillaries, which influences the diffusion signal into a voxel. With this method, IVIM determines the diffusion coefficient (D), the perfusion parameters f (perfusion fraction), D* (pseudo-diffusion coefficient), and the product fD* [33].

IVIM is capable of diagnosing a wide range of cancers, but there is restricted literature for evaluating the effectiveness of RT [34]. Kooreman et al. have published two studies assessing changes in IVIM parameters during RT in PC patients. An increased trend in D, f, and D* parameters revealed a positive response to RT unless fD* parameters remained stable during RT sessions [35,36]. IVIM parameters could be a promising biomarker of successful RT; however, further research is needed to validate this consideration. Unfortunately, IVIM still has a long way to go in clinical routine due to the absence of standard protocol and its sensitivity to acquisition parameters (b-values and TE) and segmentation method [34].

#### 1.2.4. The Role of MRI-Based Radiomics on RT Evaluation

A challenge in using MRI to assess BCR is the insufficient collaboration between radiation oncologists and radiologists, which can result in the misinterpretation of MRI scans, especially in the case of treatment-related changes, like inflammation or edema, which can reduce the accurate interpretation of images. Radiomics may overcome these limitations by providing a reliable tool for accurate predictive and objective evaluation of treatment response.

Radiomics refers to extractions of many texture features from MR images. These features are the cornerstone for developing classification and predicted models on various aspects of cancer, such as diagnosis, prognosis and response to treatment [37].

Recently, there has been a growing interest in utilizing these texture features from pretreatment MRI to predict the occurrence of BCR. Gnep et al. have found that Haralick features from T2WI were significantly associated with BCR occurrence [38]. In a similar study, Shiradkar R. et al. proved that CoLlAGe and Gabor’s features provide additional information that enhances the predictive accuracy οf BCR on PCa [39]. Identifying patients with elevated risk for BCR before treatment may offer them the benefit of alternative or more aggressive treatment options, including adjuvant therapy.

At last, recently, radiomics feature trends observed through delta-radiomics, the analysis of changes in radiomics features over time, which can provide valuable clinical insights and improve treatment response prediction in longitudinal imaging studies [37]. Abdollahi et al. investigate the predictive performance of delta-radiomics models on RT response in PCa [40]. In 33 patients pre- and post-treatment, features were extracted from T2WI and ADC images with AUCT2 = 0.68 and AUCADC 0.62. Thus, radiomics appearcapable of predicting PCa recurrence after RT, but standardization of the method is necessary.

**Table 1:** The characteristics of MRI sequences in BCR detection after RT and respective advantages and disadvantages.

Study, Year	No. of Subjects	MRI Technique	Biomarkers Change on PCaRelapse	Sensitivity	Specificity	Advantages	Disadvantages
Westphalen et al., 2009 [21]	22 patients	T2-Weighted Imaging (T2WI)	Hypointense lesion with a capsular bulge	Reader 1: 62% (95% CI: 0.45–0.76) Reader 2:74% (95% CI: 0.57–0.86)	Reader 1: 64% (95% CI: 0.45–0.80) Reader 2: 68% (95% CI: 0.49–0.82)	Localization of lesion	Background signal changes reduce the diagnostic accuracy
Koopman et al., 2020 [22]	review	Diffusion Weighted Imaging (DWI)	Increase DWI intensity and reduce ADC	Range: 94–100%	Range: 75% to 100%	High accuracy in post-treatment changes	False indications of lesions due to radiation inflammatory changes
Koopman et al., 2020 [22]	review	Dynamic contrast-enhanced DCE	Increased wash-in and wash-out patterns	Range: 70% to 74%	Range: 73% to 85%.	Key role on the treatment’s effectiveness.	Radiation causes inflammatory changes in blood volume
Liao et al., 2018 [23]	review	Magnetic Resonance Spectroscopy (MRS)	Increase concentration of choline and citrate.	69% (95% CI: 0.58–0.78)	86% (95% CI: 0.79–0.92)	Indicative biomarkers of prostate energy metabolism tumor membrane cellular activity	Long image acquisition High cost Low sensitivity
Arai et al., 2021 [24]	Animal study	Blood Oxygenation Level Dependent (BOLD) and Tissue Oxygen Level Dependent (TOLD) MRI	Decrease R2* and T1 signal.	N/A	N/A	Capable biomarkersforthe prediction of RT failure outcomes	Limited studies on animals
Kooreman et al., 2021, 2022 [34,35]	20 and 22 patients, respectively	Intravoxel Incoherent Motion (IVIM)	Decrease in D, f, and D* parameters.	N/A	N/A	Perfusion and diffusion information without the use of contrast agents	Absence of standard protocol sensitivity acquisition parameters (b-values and TE) and segmentation method.
Gnep et al., 2017 [37]	74 patients	T2WI and Radiomics Analysis	Haralick features correlated with PCa relapse	N/A	N/A	Reliable tool for accurate interpretation and objective evaluation of RT response.	Absence of standard method

## 2. Discussion

Twenty-five percent of PCa patients undergoing RT will develop BCR suggested by the increasing PSA blood levels [6]. Considering, however, the low specificity of the PSA blood test to diagnose local vs. regional and distant relapse, there is an increasing interest in a more accurate method to identify intraprostatic cancer recurrence [7,8].

Recent developments in novel imaging modalities have led to significant improvement in evaluation of patients with biochemically recurrent PCa. T2WI has a limited role in BCR detection but contributes to structural changes within the prostate after RT. DWI assessing the water movement restrictions in recurrent tumors with high b-value and low ADC-value improves the diagnostic accuracy.

DCE-MRI detects higher vascularity in tumors than irradiated tissues, making it easier to identify suspicious lesions. We should note that the DCE sequence presents a main drawback, false hypervascular areas on the prostate gland after RT derived by inflammatory changes due to radiation dose [41]. Therefore, some studies tended to develop contrast agents that encapsulated in prostate tumor cells and enhanced the MR evidence. The most studied MR contrast agents are supermagnetic iron platinum, protein-based, iron oxide nanoparticles [41,42,43]. Especially, nanoparticles contrast agents seem to have the advantage of use like radiosensitizer in RT [44]. Although these agents are very promising, they are still under investigation.

Following, MRS contributes positively to the previous techniques detecting the rapid decrease in citrate levels and higher choline levels in BCR. In addition, advanced imaging techniques like BOLD, TOLD, IVIM and radiomics analysis look like promising tools in the evaluation of PCa after RT but require additional investigation and standardization of methods.

The high performance of mpMRI in the evaluation of PCa recurrence was approved in three multi-reader studies using PI-RR system. Pecoraro M. et al. indicate a sensitivity of 71–81%, specificity of 74–93%, PPV of 71–89%, NPV of 79–86% and excellent ICC = 0.87 [16]. Bergaglio C. et al. have observed sensitivity of 59–83%, specificity of 87–100%, an accuracy of 80–88%, and lower ICC = 0.74 due to marked difference in experience among readers from 3 to 10 years [18]. Although Franco N.P et al. have comparable results with previous studies in sensitivity, specificity and PPV ranges, NPV values were lower in both PI-RR cut-off values (33–46% and 28–36%, respectively) [18]. These NPV low values may be related to the asymmetrical distribution of the study population, with 88.3% of cases (88.3%) shown to be confirmed local recurrence. This diagnostic overestimation caused by some paraphysiologic appearances of pelvic structures (i.e., urethral or penis bulb early enhancement) may mimic the presence of PCa local recurrences [18].

mpMRI has been reported to be useful for the detection of BCR after brachytherapy with high sensitivity. Certainly, in practice, MR detection of recurrence may misinterpret the cause of brachytherapy seeds, which may be plagued by metallic artifacts as well as other limitations such as inflammatory changes [45].

Additionally, there is evidence that mpMRI could evaluate PCa recurrence after minimally invasive alternative therapeutic modalities such as high-intensity focused ultrasound (HIFU), cryotherapeutic ablation and focal photodynamic therapy (PDT), radiofrequency ablation (RFA) and irreversible electroporation [46]. Nevertheless, the adverse and long-term side-effects of the above therapies are under investigation and are not included in standard management guidelines for PCa [2,46].

In recent years, [68Ga] PSMA-11 PET-CT and [68Ga] PSMA-11 PET-MRI have approved efficient diagnostic methods for detecting PCa local recurrence. However, the ligand [68Ga] PSMA-11 released and led to high radioactivity accumulation in the urinary bladder [47,48]. This radiation aggregation may cover the detection of PCa local recurrence leading to impaired assessment. In the other hand, PET-CT or PET-MRI have the potential to identify small pathological pelvis or distant lymph nodes, which ability even whole-body MRI does not have [49]. PET-MRI has not yet been applied in the clinical management of PCa [50]. Therefore, mp-MRI of the pelvis is considered a cornerstone in local PCa failure.

The present study has some limitations. In theory, particle beam radiation therapy such as protons or neutrons are attractive alternatives in RT for PCa. However, information on proton and neutron therapies is still limited. Especially, protons’ therapy studies from the SEER database and from Harvard describe higher toxicity than photon therapy in the genitourinary system [2]. So, this study focuses only on external beam photon therapy.

Another severe limitation of this study is MR assessment of PCa recurrence after RT and androgen deprivation therapy (ADT) [51]. ADT reduces glandular prostate tissues and suppresses any potential tumor regrowth, leading to less obvious visual lesions on T2-weighted images. Furthermore, it decreases cellular density and vascularity, reducing the sensitivity of the DWI and DCE sequences causing lower or absent enhancement in both normal and recurrent tissue. Finally, it minimizes the glandular activity and alters the metabolic profile reflected in MRS. As consequences, the RT- and ADT-induced changes often obscure accurate imaging [51].

## 3. Conclusions

The continuous advancement of MRI techniques, along with radiomics, is opening new possibilities in the effectiveness of RT for PCa. The high levels of sensitivity and specificity demonstrated by these imaging techniques indicate their necessity in follow-up protocols, particularly in the case of PSA failure after RT. Radiation oncologists and radiologists need to work together and integrate these emerging techniques into clinical practice. Staying abreast of medical and technological developments is critical for enhancing the quality of patient care and their quality of life.
